# Crystal structure of 2-((1*E*)-{2-[bis­(2-methyl­benzyl­sulfan­yl)methyl­idene]hydrazin-1-yl­idene}meth­yl)-6-meth­oxy­phenol

**DOI:** 10.1107/S2056989015004946

**Published:** 2015-03-18

**Authors:** Enis Nadia Md Yusof, Thahira Begum S. A. Ravoof, Mohamed Ibrahim Mohamed Tahir, Edward R. T. Tiekink

**Affiliations:** a Department of Chemistry, Universiti Putra Malaysia, 43400 Serdang, Malaysia; b Department of Chemistry, University of Malaya, 50603 Kuala Lumpur, Malaysia

**Keywords:** crystal structure, *S*-substituted di­thio­carbaza­tes, hydrogen bonding, C—H⋯π inter­actions, π–π inter­actions

## Abstract

In the title compound, C_25_H_26_N_2_O_2_S_2_, the central CN_2_S_2_ atoms are almost coplanar (r.m.s. deviation = 0.0058 Å). One phenyl ring clearly lies to one side of the central plane, while the other is oriented in the plane but splayed. Despite the different relative orientations, the phenyl rings form similar dihedral angles of 64.90 (3) and 70.06 (3)° with the central plane, and 63.28 (4)° with each other. The benzene ring is twisted with respect to the central plane, forming a dihedral angle of 13.17 (7)°. The S_2_C=N, N—N and N—N=C bond lengths of 1.2919 (19), 1.4037 (17) and 1.2892 (19) Å, respectively, suggest limited conjugation over these atoms; the configuration about the N—N=C bond is *E*. An intra­molecular O—H⋯N hydrogen bond is noted. In the crystal, phen­yl–meth­oxy C—H⋯O and phen­yl–phenyl C—H⋯π inter­actions lead to supra­molecular double chains parallel to the *b* axis. These are connected into a layer *via* meth­yl–phenyl C—H⋯π inter­actions, and layers stack along the *a* axis, being connected by weak π–π inter­actions between phenyl rings [inter-centroid distance = 3.9915 (9) Å] so that a three-dimensional architecture ensues.

## Related literature   

For background to the coordination chemistry of di­thio­carbazate derivatives, see: Tarafder *et al.* (2002 ▸); Ravoof *et al.* (2010 ▸, 2011 ▸); Omar *et al.* (2014 ▸). For related synthesis, see: Ali & Tarafder (1977 ▸); Tarafder *et al.* (2002 ▸); Manan *et al.* (2012 ▸). For a related structure but with the S atoms connected by an ethyl­ene bridge, and with a terminal furan-2-yl ring, *i.e. N*-1,3-di­thio­lan-2-yl­idene-*N*′-[(*E*)-furan-2-yl­methyl­idene]hydrazone, see: Liu *et al.* (2008 ▸).
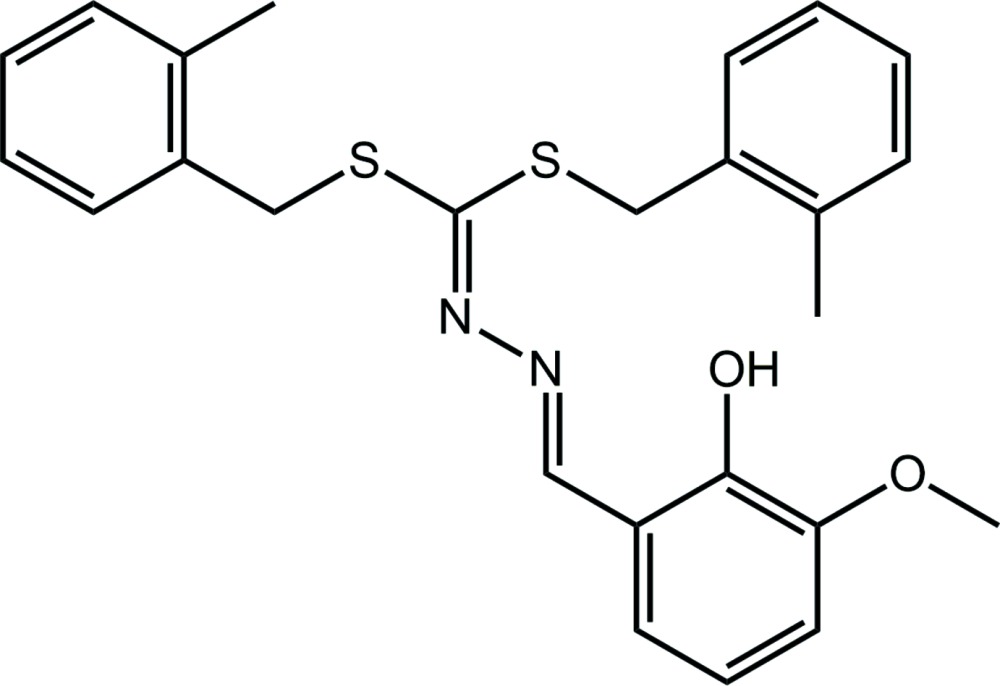


## Experimental   

### Crystal data  

C_25_H_26_N_2_O_2_S_2_
*M* *_r_* = 450.60
Monoclinic, 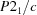

*a* = 19.7865 (7) Å
*b* = 6.8600 (2) Å
*c* = 16.1805 (5) Å
β = 94.880 (3)°
*V* = 2188.31 (12) Å^3^
*Z* = 4
Cu *K*α radiation
μ = 2.41 mm^−1^
*T* = 100 K
0.25 × 0.11 × 0.08 mm

### Data collection  

Oxford Diffraction Xcaliber Eos Gemini diffractometer
Absorption correction: multi-scan (*CrysAlis PRO*; Agilent, 2011 ▸) *T*_min_ = 0.715, *T*_max_ = 1.000
40860 measured reflections
4248 independent reflections
3956 reflections with *I* > 2σ(*I*)
*R*_int_ = 0.033

### Refinement  

*R*[*F* ^2^ > 2σ(*F* ^2^)] = 0.035
*wR*(*F* ^2^) = 0.094
*S* = 1.04
4248 reflections
286 parameters
1 restraint
H atoms treated by a mixture of independent and constrained refinement
Δρ_max_ = 0.41 e Å^−3^
Δρ_min_ = −0.24 e Å^−3^

### 

Data collection: *CrysAlis PRO* (Agilent, 2011 ▸); cell refinement: *CrysAlis PRO*; data reduction: *CrysAlis PRO*; program(s) used to solve structure: *SHELXS97* (Sheldrick, 2015 ▸); program(s) used to refine structure: *SHELXL2014* (Sheldrick, 2015 ▸); molecular graphics: *ORTEP-3 for Windows* (Farrugia, 2012 ▸) and *DIAMOND* (Brandenburg, 2006 ▸); software used to prepare material for publication: *publCIF* (Westrip, 2010 ▸).

## Supplementary Material

Crystal structure: contains datablock(s) ml, I. DOI: 10.1107/S2056989015004946/hb7379sup1.cif

Structure factors: contains datablock(s) I. DOI: 10.1107/S2056989015004946/hb7379Isup2.hkl

Supporting information file. DOI: 10.1107/S2056989015004946/hb7379Isup3.cml

**Fig. 1.**
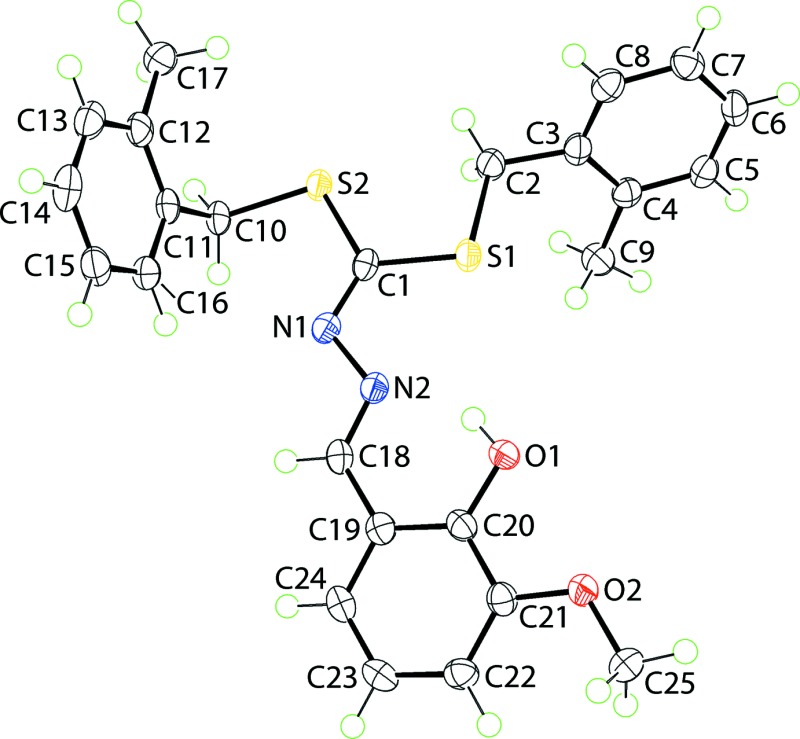
The molecular structure of the title compound showing displacement ellipsoids at the 70% probability level.

**Fig. 2.**
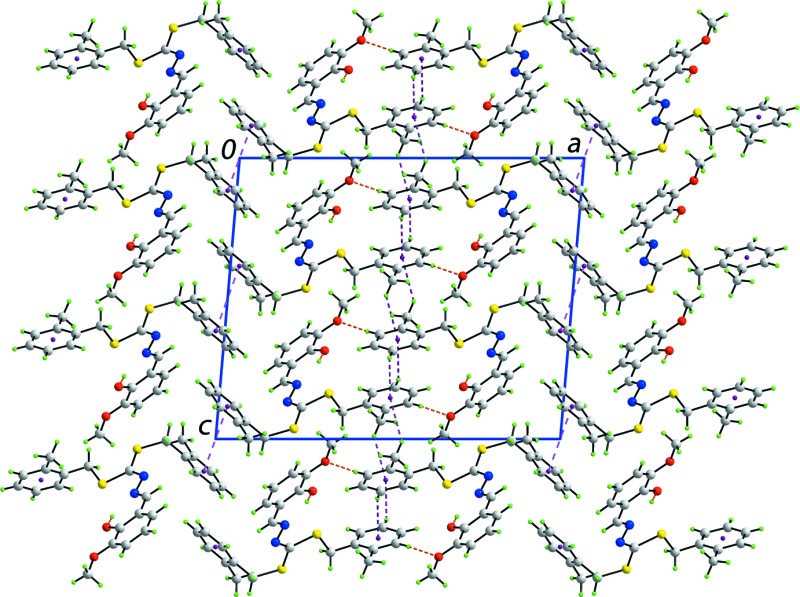
A view of the unit-cell contents in projection down the b axis. The C—H···O, C—H···π and π—π interactions are shown as orange, purple and pink dashed lines, respectively.

. DOI: 10.1107/S2056989015004946/hb7379fig1.tif
The mol­ecular structure of the title compound showing displacement ellipsoids at the 70% probability level.

b . DOI: 10.1107/S2056989015004946/hb7379fig2.tif
A view of the unit-cell contents in projection down the *b* axis. The C—H⋯O, C—H⋯π and π—π inter­actions are shown as orange, purple and pink dashed lines, respectively.

CCDC reference: 1053188

Additional supporting information:  crystallographic information; 3D view; checkCIF report

## Figures and Tables

**Table 1:** Hydrogen-bond geometry (, ) *Cg*1 is the centroid of the C3C8 ring.

*D*H*A*	*D*H	H*A*	*D* *A*	*D*H*A*
O1H1*O*N2	0.84(2)	1.87(2)	2.6331(16)	151(2)
C5H5O2^i^	0.95	2.44	3.3868(18)	176
C7H7*Cg*1^i^	0.95	2.68	3.5046(15)	146
C9H9*B* *Cg*1^ii^	0.98	2.72	3.5482(17)	142

Symmetry codes: (i) 
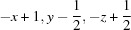
; (ii) 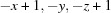.

## supplementary crystallographic information

### S1. Experimental

The synthesis of *S*-2-methylbenzyldithiocarbazate (S2MBDTC) was accomplished as reported previously (Ravoof *et al.*, 2011). The title compound was synthesized following an established literature procedure (Ravoof *et al.*, 2011). S2MBDTC (2.12.g, 0.01 mol) was dissolved in hot acetonitrile (150 ml). This was added to an equimolar solution of 2-hydroxy-3-methoxybenzaldehyde (1.52 g, 0.01 mol) in ethanol (20 ml). The mixture was heated and stirred for 30 min and then allowed to stand for a few hours. The yellow crystals formed were filtered off and recrystallized from acetonitrile. Pale-yellow plates were obtained after 1 week by keeping the solution at room temperature.

### S2. Refinement

Carbon-bound H-atoms were placed in calculated positions (C—H = 0.95 to 0.99 Å) and were included in the refinement in the riding model approximation with *U_iso_*(H) = 1.2*U_eq_*(C). The O—H H atom was refined with O—H = 0.84±0.01 Å, and with *U_iso_*(H) = 1.5*U_eq_*(O).

### Crystal data

**Table:**

C_25_H_26_N_2_O_2_S_2_	*F*(000) = 952
*M**_r_* = 450.60	*D*_x_ = 1.368 Mg m^−^^3^
Monoclinic, *P*2_1_/*c*	Cu *K*α radiation, λ = 1.5418 Å
*a* = 19.7865 (7) Å	Cell parameters from 17455 reflections
*b* = 6.8600 (2) Å	θ = 3.4–71.3°
*c* = 16.1805 (5) Å	µ = 2.41 mm^−^^1^
β = 94.880 (3)°	*T* = 100 K
*V* = 2188.31 (12) Å^3^	Plate, pale-yellow
*Z* = 4	0.25 × 0.11 × 0.08 mm

### Data collection

**Table:**

Oxford Diffraction Xcaliber Eos Gemini diffractometer	4248 independent reflections
Radiation source: fine-focus sealed tube	3956 reflections with *I* > 2σ(*I*)
Graphite monochromator	*R*_int_ = 0.033
Detector resolution: 16.1952 pixels mm^-1^	θ_max_ = 71.4°, θ_min_ = 4.5°
ω scans	*h* = −24→24
Absorption correction: multi-scan (*CrysAlis PRO*; Agilent, 2011)	*k* = −8→8
*T*_min_ = 0.715, *T*_max_ = 1.000	*l* = −19→19
40860 measured reflections	

### Refinement

**Table:**

Refinement on *F*^2^	1 restraint
Least-squares matrix: full	Hydrogen site location: mixed
*R*[*F*^2^ > 2σ(*F*^2^)] = 0.035	H atoms treated by a mixture of independent and constrained refinement
*wR*(*F*^2^) = 0.094	*w* = 1/[σ^2^(*F*_o_^2^) + (0.0589*P*)^2^ + 0.9765*P*] where *P* = (*F*_o_^2^ + 2*F*_c_^2^)/3
*S* = 1.04	(Δ/σ)_max_ = 0.001
4248 reflections	Δρ_max_ = 0.41 e Å^−^^3^
286 parameters	Δρ_min_ = −0.24 e Å^−^^3^

### Special details

**Table:**

Geometry. All e.s.d.'s (except the e.s.d. in the dihedral angle between two l.s. planes) are estimated using the full covariance matrix. The cell e.s.d.'s are taken into account individually in the estimation of e.s.d.'s in distances, angles and torsion angles; correlations between e.s.d.'s in cell parameters are only used when they are defined by crystal symmetry. An approximate (isotropic) treatment of cell e.s.d.'s is used for estimating e.s.d.'s involving l.s. planes.

### Fractional atomic coordinates and isotropic or equivalent isotropic displacement parameters (Å^2^)

**Table:**

	*x*	*y*	*z*	*U*_iso_*/*U*_eq_	
S1	0.31760 (2)	0.15598 (5)	0.33906 (2)	0.01851 (11)	
S2	0.22443 (2)	0.01673 (5)	0.46350 (2)	0.01756 (11)	
O1	0.29427 (6)	0.56093 (16)	0.18572 (7)	0.0234 (2)	
H1O	0.2807 (10)	0.492 (3)	0.2236 (10)	0.035*	
O2	0.32372 (5)	0.83198 (15)	0.08026 (7)	0.0220 (2)	
N1	0.19930 (6)	0.32696 (18)	0.36803 (7)	0.0176 (3)	
N2	0.22172 (6)	0.44896 (18)	0.30629 (7)	0.0173 (3)	
C1	0.24117 (7)	0.1860 (2)	0.38658 (8)	0.0159 (3)	
C2	0.36133 (8)	−0.0340 (2)	0.40266 (10)	0.0219 (3)	
H2A	0.3653	0.0069	0.4615	0.026*	
H2B	0.3345	−0.1559	0.3981	0.026*	
C3	0.43130 (7)	−0.0713 (2)	0.37492 (9)	0.0184 (3)	
C4	0.48507 (8)	0.0607 (2)	0.39319 (9)	0.0179 (3)	
C5	0.54953 (8)	0.0092 (2)	0.37141 (9)	0.0191 (3)	
H5	0.5862	0.0971	0.3829	0.023*	
C6	0.56105 (8)	−0.1674 (2)	0.33335 (9)	0.0205 (3)	
H6	0.6055	−0.2002	0.3200	0.025*	
C7	0.50798 (8)	−0.2964 (2)	0.31473 (9)	0.0214 (3)	
H7	0.5157	−0.4175	0.2886	0.026*	
C8	0.44318 (8)	−0.2458 (2)	0.33495 (9)	0.0210 (3)	
H8	0.4064	−0.3322	0.3212	0.025*	
C9	0.47480 (8)	0.2536 (2)	0.43450 (10)	0.0229 (3)	
H9A	0.5178	0.3254	0.4400	0.034*	
H9B	0.4592	0.2315	0.4896	0.034*	
H9C	0.4407	0.3296	0.4008	0.034*	
C10	0.14184 (7)	0.0968 (2)	0.49331 (9)	0.0180 (3)	
H10A	0.1365	0.0506	0.5503	0.022*	
H10B	0.1411	0.2411	0.4943	0.022*	
C11	0.08218 (7)	0.0259 (2)	0.43695 (9)	0.0180 (3)	
C12	0.06032 (7)	−0.1688 (2)	0.43911 (9)	0.0189 (3)	
C13	0.00459 (8)	−0.2245 (2)	0.38554 (9)	0.0228 (3)	
H13	−0.0107	−0.3558	0.3861	0.027*	
C14	−0.02900 (8)	−0.0927 (3)	0.33158 (10)	0.0249 (3)	
H14	−0.0664	−0.1344	0.2952	0.030*	
C15	−0.00799 (8)	0.1002 (2)	0.33070 (9)	0.0232 (3)	
H15	−0.0314	0.1917	0.2947	0.028*	
C16	0.04763 (8)	0.1581 (2)	0.38300 (9)	0.0201 (3)	
H16	0.0624	0.2897	0.3821	0.024*	
C17	0.09534 (8)	−0.3151 (2)	0.49745 (9)	0.0227 (3)	
H17A	0.0692	−0.4366	0.4955	0.034*	
H17B	0.0987	−0.2631	0.5540	0.034*	
H17C	0.1410	−0.3409	0.4808	0.034*	
C18	0.18647 (7)	0.6061 (2)	0.29468 (9)	0.0171 (3)	
H18	0.1490	0.6274	0.3267	0.020*	
C19	0.20239 (7)	0.7516 (2)	0.23404 (8)	0.0168 (3)	
C20	0.25580 (7)	0.7241 (2)	0.18293 (9)	0.0178 (3)	
C21	0.27077 (7)	0.8714 (2)	0.12674 (9)	0.0179 (3)	
C22	0.23361 (8)	1.0427 (2)	0.12261 (9)	0.0193 (3)	
H22	0.2443	1.1429	0.0853	0.023*	
C23	0.18044 (8)	1.0686 (2)	0.17308 (9)	0.0203 (3)	
H23	0.1549	1.1860	0.1694	0.024*	
C24	0.16474 (7)	0.9262 (2)	0.22791 (9)	0.0190 (3)	
H24	0.1284	0.9453	0.2618	0.023*	
C25	0.33745 (8)	0.9728 (2)	0.01891 (10)	0.0223 (3)	
H25A	0.2976	0.9870	−0.0209	0.034*	
H25B	0.3762	0.9296	−0.0103	0.034*	
H25C	0.3480	1.0985	0.0458	0.034*	

### Atomic displacement parameters (Å^2^)

**Table:**

	*U*^11^	*U*^22^	*U*^33^	*U*^12^	*U*^13^	*U*^23^
S1	0.01353 (19)	0.0233 (2)	0.01915 (19)	0.00182 (13)	0.00389 (13)	0.00516 (13)
S2	0.01378 (19)	0.02052 (19)	0.01875 (19)	0.00014 (13)	0.00349 (13)	0.00367 (13)
O1	0.0240 (6)	0.0197 (5)	0.0281 (6)	0.0072 (4)	0.0118 (5)	0.0080 (5)
O2	0.0203 (5)	0.0228 (5)	0.0240 (5)	0.0052 (4)	0.0079 (4)	0.0076 (4)
N1	0.0168 (6)	0.0180 (6)	0.0184 (6)	−0.0019 (5)	0.0030 (5)	0.0006 (5)
N2	0.0157 (6)	0.0186 (6)	0.0179 (6)	−0.0006 (5)	0.0030 (5)	0.0015 (5)
C1	0.0133 (7)	0.0191 (7)	0.0155 (6)	−0.0036 (5)	0.0018 (5)	−0.0015 (5)
C2	0.0162 (7)	0.0242 (8)	0.0257 (8)	0.0031 (6)	0.0036 (6)	0.0093 (6)
C3	0.0150 (7)	0.0232 (7)	0.0171 (7)	0.0017 (6)	0.0018 (5)	0.0063 (6)
C4	0.0190 (7)	0.0200 (7)	0.0149 (6)	0.0005 (6)	0.0020 (5)	0.0033 (5)
C5	0.0165 (7)	0.0234 (8)	0.0174 (7)	−0.0019 (6)	0.0016 (6)	0.0038 (6)
C6	0.0163 (7)	0.0281 (8)	0.0178 (7)	0.0037 (6)	0.0047 (6)	0.0039 (6)
C7	0.0246 (8)	0.0217 (7)	0.0182 (7)	0.0026 (6)	0.0034 (6)	−0.0007 (6)
C8	0.0193 (7)	0.0222 (7)	0.0210 (7)	−0.0030 (6)	−0.0002 (6)	0.0020 (6)
C9	0.0223 (8)	0.0225 (8)	0.0239 (7)	0.0012 (6)	0.0015 (6)	−0.0004 (6)
C10	0.0144 (7)	0.0224 (7)	0.0180 (7)	−0.0001 (6)	0.0057 (5)	−0.0016 (6)
C11	0.0137 (7)	0.0234 (7)	0.0178 (7)	0.0010 (6)	0.0069 (5)	−0.0021 (6)
C12	0.0173 (7)	0.0230 (7)	0.0175 (7)	0.0016 (6)	0.0077 (6)	−0.0006 (6)
C13	0.0202 (8)	0.0249 (8)	0.0243 (8)	−0.0039 (6)	0.0076 (6)	−0.0024 (6)
C14	0.0150 (7)	0.0377 (9)	0.0223 (7)	−0.0048 (7)	0.0032 (6)	−0.0017 (7)
C15	0.0171 (7)	0.0309 (8)	0.0223 (7)	0.0046 (6)	0.0051 (6)	0.0053 (6)
C16	0.0178 (7)	0.0208 (7)	0.0228 (7)	0.0008 (6)	0.0075 (6)	0.0007 (6)
C17	0.0238 (8)	0.0217 (7)	0.0233 (7)	−0.0002 (6)	0.0054 (6)	0.0020 (6)
C18	0.0131 (7)	0.0213 (7)	0.0168 (7)	−0.0009 (5)	0.0017 (5)	−0.0029 (6)
C19	0.0152 (7)	0.0183 (7)	0.0164 (7)	−0.0007 (5)	−0.0004 (5)	−0.0020 (5)
C20	0.0150 (7)	0.0178 (7)	0.0202 (7)	0.0022 (5)	0.0001 (5)	−0.0006 (6)
C21	0.0156 (7)	0.0206 (7)	0.0174 (7)	0.0007 (6)	0.0004 (5)	−0.0004 (6)
C22	0.0198 (7)	0.0190 (7)	0.0185 (7)	0.0007 (6)	−0.0012 (6)	0.0031 (6)
C23	0.0198 (7)	0.0182 (7)	0.0224 (7)	0.0050 (6)	−0.0022 (6)	−0.0015 (6)
C24	0.0148 (7)	0.0227 (8)	0.0192 (7)	0.0025 (6)	0.0010 (5)	−0.0037 (6)
C25	0.0237 (8)	0.0219 (8)	0.0221 (7)	−0.0005 (6)	0.0056 (6)	0.0048 (6)

### Geometric parameters (Å, º)

**Table:**

S1—C1	1.7658 (14)	C10—H10B	0.9900
S1—C2	1.8317 (15)	C11—C16	1.396 (2)
S2—C1	1.7543 (14)	C11—C12	1.405 (2)
S2—C10	1.8271 (14)	C12—C13	1.397 (2)
O1—C20	1.3519 (18)	C12—C17	1.506 (2)
O1—H1O	0.835 (9)	C13—C14	1.387 (2)
O2—C21	1.3676 (18)	C13—H13	0.9500
O2—C25	1.4279 (18)	C14—C15	1.387 (2)
N1—C1	1.2919 (19)	C14—H14	0.9500
N1—N2	1.4037 (17)	C15—C16	1.388 (2)
N2—C18	1.2892 (19)	C15—H15	0.9500
C2—C3	1.513 (2)	C16—H16	0.9500
C2—H2A	0.9900	C17—H17A	0.9800
C2—H2B	0.9900	C17—H17B	0.9800
C3—C8	1.390 (2)	C17—H17C	0.9800
C3—C4	1.409 (2)	C18—C19	1.453 (2)
C4—C5	1.397 (2)	C18—H18	0.9500
C4—C9	1.504 (2)	C19—C20	1.409 (2)
C5—C6	1.387 (2)	C19—C24	1.409 (2)
C5—H5	0.9500	C20—C21	1.408 (2)
C6—C7	1.387 (2)	C21—C22	1.385 (2)
C6—H6	0.9500	C22—C23	1.397 (2)
C7—C8	1.394 (2)	C22—H22	0.9500
C7—H7	0.9500	C23—C24	1.373 (2)
C8—H8	0.9500	C23—H23	0.9500
C9—H9A	0.9800	C24—H24	0.9500
C9—H9B	0.9800	C25—H25A	0.9800
C9—H9C	0.9800	C25—H25B	0.9800
C10—C11	1.509 (2)	C25—H25C	0.9800
C10—H10A	0.9900		
			
C1—S1—C2	102.68 (7)	C13—C12—C11	118.25 (14)
C1—S2—C10	102.44 (7)	C13—C12—C17	120.22 (14)
C20—O1—H1O	105.9 (14)	C11—C12—C17	121.53 (14)
C21—O2—C25	116.65 (11)	C14—C13—C12	121.52 (15)
C1—N1—N2	112.11 (12)	C14—C13—H13	119.2
C18—N2—N1	113.74 (12)	C12—C13—H13	119.2
N1—C1—S2	120.28 (11)	C13—C14—C15	120.04 (14)
N1—C1—S1	122.83 (11)	C13—C14—H14	120.0
S2—C1—S1	116.88 (8)	C15—C14—H14	120.0
C3—C2—S1	110.74 (10)	C14—C15—C16	119.30 (14)
C3—C2—H2A	109.5	C14—C15—H15	120.3
S1—C2—H2A	109.5	C16—C15—H15	120.3
C3—C2—H2B	109.5	C15—C16—C11	121.05 (14)
S1—C2—H2B	109.5	C15—C16—H16	119.5
H2A—C2—H2B	108.1	C11—C16—H16	119.5
C8—C3—C4	119.77 (13)	C12—C17—H17A	109.5
C8—C3—C2	118.61 (13)	C12—C17—H17B	109.5
C4—C3—C2	121.50 (14)	H17A—C17—H17B	109.5
C5—C4—C3	118.28 (14)	C12—C17—H17C	109.5
C5—C4—C9	119.68 (13)	H17A—C17—H17C	109.5
C3—C4—C9	122.04 (13)	H17B—C17—H17C	109.5
C6—C5—C4	121.39 (14)	N2—C18—C19	121.81 (13)
C6—C5—H5	119.3	N2—C18—H18	119.1
C4—C5—H5	119.3	C19—C18—H18	119.1
C7—C6—C5	120.27 (14)	C20—C19—C24	119.42 (13)
C7—C6—H6	119.9	C20—C19—C18	121.40 (13)
C5—C6—H6	119.9	C24—C19—C18	119.16 (13)
C6—C7—C8	119.00 (14)	O1—C20—C21	117.85 (13)
C6—C7—H7	120.5	O1—C20—C19	122.66 (13)
C8—C7—H7	120.5	C21—C20—C19	119.49 (13)
C3—C8—C7	121.26 (14)	O2—C21—C22	124.72 (13)
C3—C8—H8	119.4	O2—C21—C20	115.23 (13)
C7—C8—H8	119.4	C22—C21—C20	120.03 (13)
C4—C9—H9A	109.5	C21—C22—C23	120.19 (14)
C4—C9—H9B	109.5	C21—C22—H22	119.9
H9A—C9—H9B	109.5	C23—C22—H22	119.9
C4—C9—H9C	109.5	C24—C23—C22	120.68 (14)
H9A—C9—H9C	109.5	C24—C23—H23	119.7
H9B—C9—H9C	109.5	C22—C23—H23	119.7
C11—C10—S2	114.51 (10)	C23—C24—C19	120.18 (13)
C11—C10—H10A	108.6	C23—C24—H24	119.9
S2—C10—H10A	108.6	C19—C24—H24	119.9
C11—C10—H10B	108.6	O2—C25—H25A	109.5
S2—C10—H10B	108.6	O2—C25—H25B	109.5
H10A—C10—H10B	107.6	H25A—C25—H25B	109.5
C16—C11—C12	119.83 (14)	O2—C25—H25C	109.5
C16—C11—C10	118.98 (14)	H25A—C25—H25C	109.5
C12—C11—C10	121.18 (13)	H25B—C25—H25C	109.5
			
C1—N1—N2—C18	−170.55 (13)	C10—C11—C12—C17	−0.1 (2)
N2—N1—C1—S2	179.01 (10)	C11—C12—C13—C14	−0.3 (2)
N2—N1—C1—S1	0.46 (17)	C17—C12—C13—C14	179.51 (14)
C10—S2—C1—N1	2.58 (13)	C12—C13—C14—C15	−0.8 (2)
C10—S2—C1—S1	−178.79 (8)	C13—C14—C15—C16	1.3 (2)
C2—S1—C1—N1	169.89 (12)	C14—C15—C16—C11	−0.7 (2)
C2—S1—C1—S2	−8.71 (10)	C12—C11—C16—C15	−0.4 (2)
C1—S1—C2—C3	−176.06 (11)	C10—C11—C16—C15	−179.26 (13)
S1—C2—C3—C8	−109.37 (14)	N1—N2—C18—C19	179.27 (12)
S1—C2—C3—C4	74.54 (16)	N2—C18—C19—C20	2.3 (2)
C8—C3—C4—C5	−1.1 (2)	N2—C18—C19—C24	−175.95 (13)
C2—C3—C4—C5	174.96 (13)	C24—C19—C20—O1	179.65 (13)
C8—C3—C4—C9	178.52 (13)	C18—C19—C20—O1	1.4 (2)
C2—C3—C4—C9	−5.4 (2)	C24—C19—C20—C21	0.1 (2)
C3—C4—C5—C6	−0.5 (2)	C18—C19—C20—C21	−178.11 (13)
C9—C4—C5—C6	179.90 (13)	C25—O2—C21—C22	−5.1 (2)
C4—C5—C6—C7	1.1 (2)	C25—O2—C21—C20	176.10 (13)
C5—C6—C7—C8	−0.1 (2)	O1—C20—C21—O2	0.0 (2)
C4—C3—C8—C7	2.1 (2)	C19—C20—C21—O2	179.55 (12)
C2—C3—C8—C7	−174.06 (13)	O1—C20—C21—C22	−178.84 (13)
C6—C7—C8—C3	−1.5 (2)	C19—C20—C21—C22	0.7 (2)
C1—S2—C10—C11	82.27 (12)	O2—C21—C22—C23	−179.84 (14)
S2—C10—C11—C16	−106.83 (14)	C20—C21—C22—C23	−1.1 (2)
S2—C10—C11—C12	74.37 (15)	C21—C22—C23—C24	0.7 (2)
C16—C11—C12—C13	0.9 (2)	C22—C23—C24—C19	0.2 (2)
C10—C11—C12—C13	179.73 (13)	C20—C19—C24—C23	−0.6 (2)
C16—C11—C12—C17	−178.88 (13)	C18—C19—C24—C23	177.71 (13)

### Hydrogen-bond geometry (Å, º)

Cg1 is the centroid of the C3–C8 ring.

**Table:**

*D*—H···*A*	*D*—H	H···*A*	*D*···*A*	*D*—H···*A*
O1—H1*O*···N2	0.84 (2)	1.87 (2)	2.6331 (16)	151 (2)
C5—H5···O2^i^	0.95	2.44	3.3868 (18)	176
C7—H7···*Cg*1^i^	0.95	2.68	3.5046 (15)	146
C9—H9*B*···*Cg*1^ii^	0.98	2.72	3.5482 (17)	142

Symmetry codes: (i) −*x*+1, *y*−1/2, −*z*+1/2; (ii) −*x*+1, −*y*, −*z*+1.
