# Supplementary figures and images for: Crystal structure of 2-((1E)-{2-[bis­(2-methyl­benzyl­sulfan­yl)methyl­idene]hydrazin-1-yl­idene}meth­yl)-6-meth­oxy­phenol
Source: Acta Crystallogr E Crystallogr Commun. 2015 Mar 18;71(Pt 4):o242–3. doi: 10.1107/S2056989015004946 (PMC4438809; doi:10.1107/S2056989015004946)

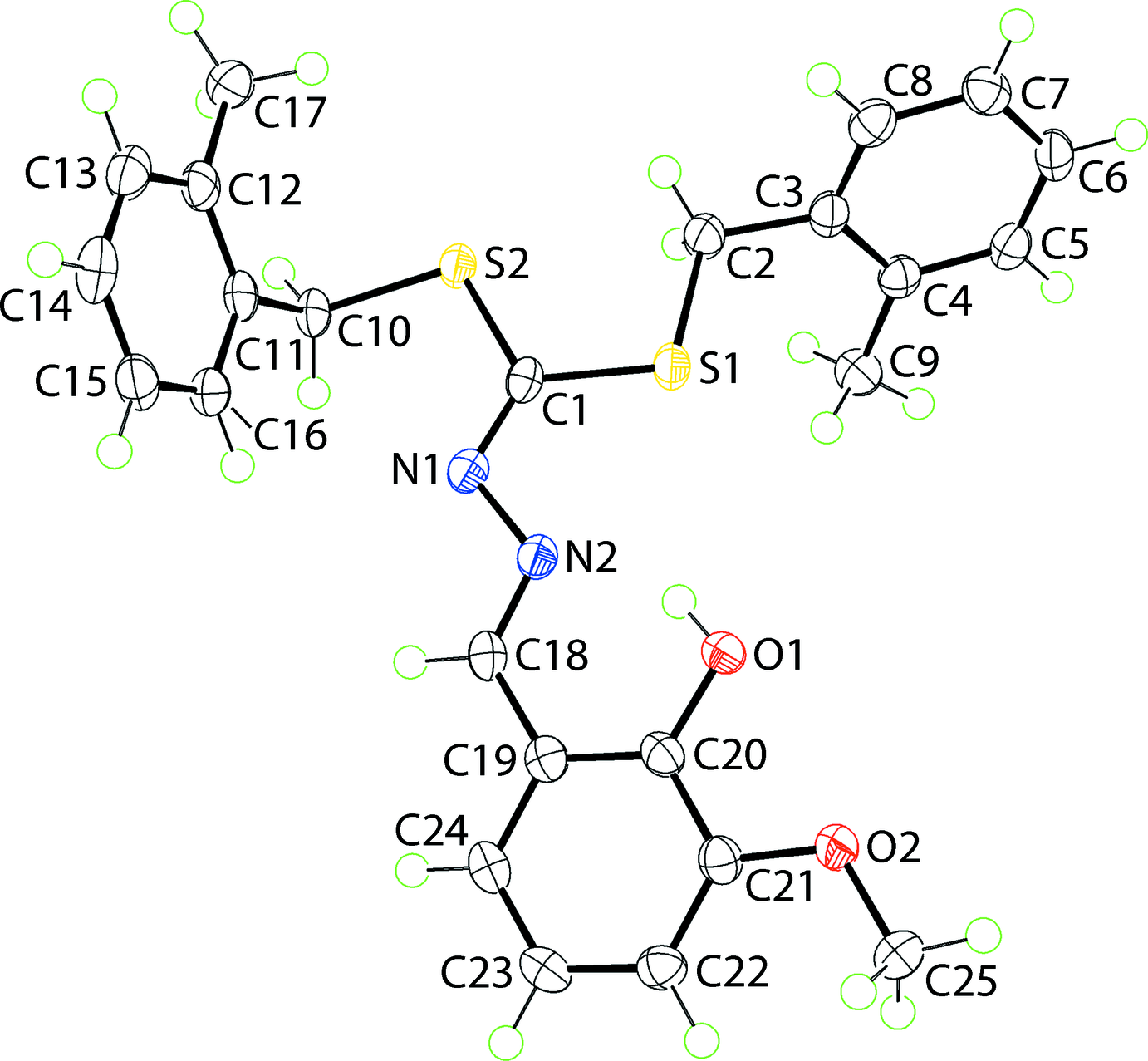

Supplement: Supplementary file 4 [file e-71-0o242-fig1.tif]

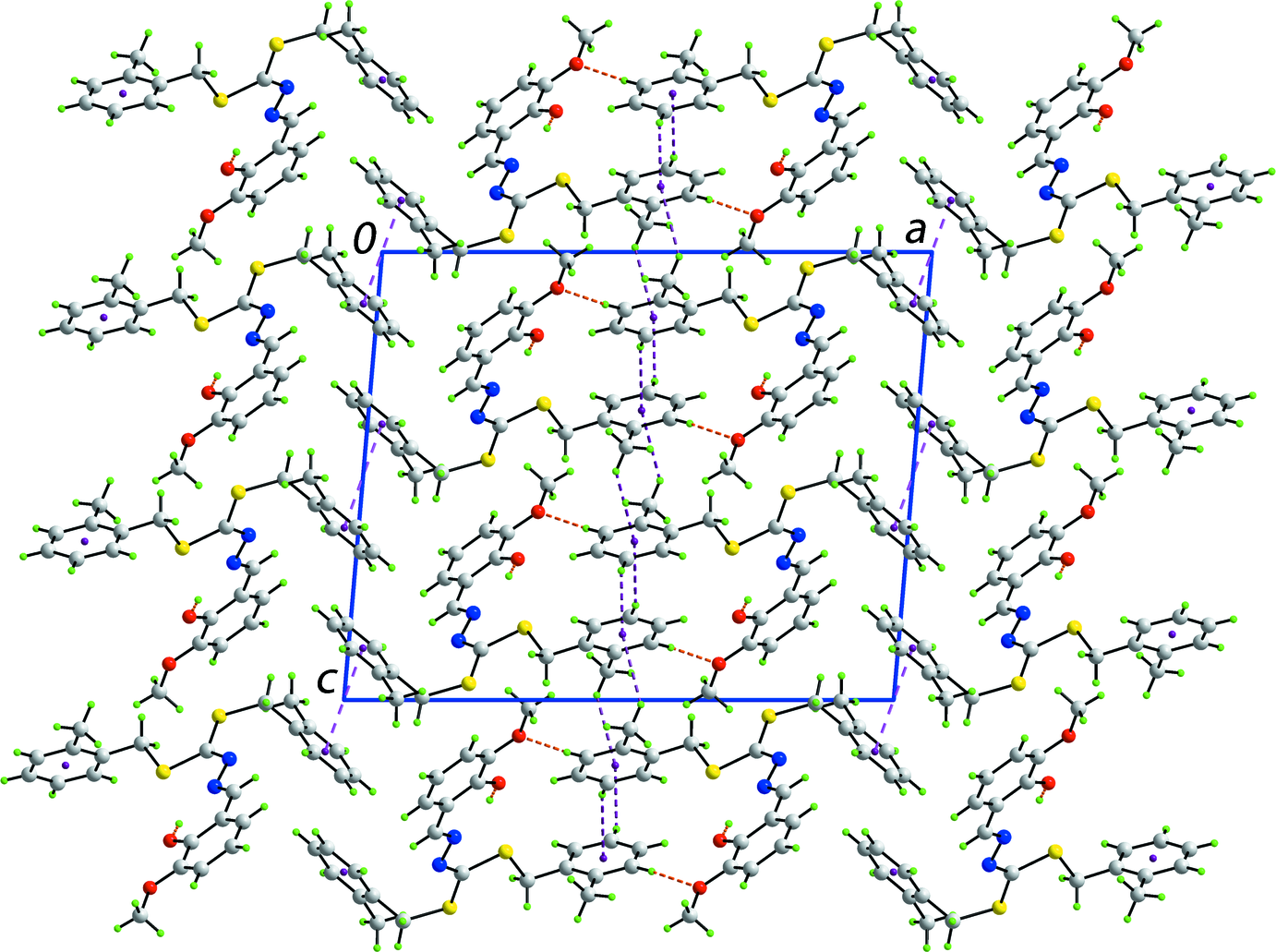

Supplement: Supplementary file 5 [file e-71-0o242-fig2.tif]
